# Editorial: Impact and consequences of COVID-19 on the musculoskeletal system

**DOI:** 10.3389/fmed.2023.1288778

**Published:** 2023-09-22

**Authors:** Eric Toussirot, Melania Maglio, Milena Fini, Francesca Salamanna

**Affiliations:** ^1^Université de Franche Comté, INSERM CIC-1431, Service de Rhumatologie, INSERM UMR Right, CHU de Besançon, Besançon, France; ^2^Surgical Sciences and Technologies, IRCCS Istituto Ortopedico Rizzoli, Bologna, Italy; ^3^IRCCS Istituto Ortopedico Rizzoli, Bologna, Italy; ^4^Surgical Sciences and Technologies, IRCCS Istituto Ortopedico Rizzoli, Bologna, Italy

**Keywords:** COVID-19, SARS-CoV-2, myalgia, viral arthritis, long COVID-19, musculoskeletal system

COVID-19 is a novel disease caused by a new strain of coronavirus called Severe Acute Respiratory Syndrome Coronavirus 2 (SARS-CoV-2). COVID-19 was first described in patients from China in December 2019, and it rapidly became evident in the first months of 2020 that the disease was spreading dramatically around the world, leading to a major global pandemic situation. The first described symptoms of COVID-19 involved the lungs, with respiratory symptoms as the leading manifestation, ranging from acute fever, cough, dyspnea to mild pneumonia or severe illness with respiratory distress (1). With the evolution of the pandemic and the emergence of new strains of the virus, there was growing evidence that the virus could induce various clinical manifestations, such as gastrointestinal, cardiovascular, neurological, dermatological, hematological, and musculoskeletal symptoms (2–4). The musculoskeletal system may be affected during the different phases of COVID-19, as an initial presentation or later, with symptoms that may persist for weeks or months after the end of the infection (5). Various rheumatologic symptoms have been described in SARS-CoV-2 that include fatigue and myalgia, arthralgia, arthritis, or the development of autoantibodies with or without autoimmune disease (6, 7). The concept of post-viral or reactive arthritis has been used to describe certain clinical features of arthritis associated with COVID-19 (8). Several explanations have been proposed to account for these various musculoskeletal complaints, and one leading reason is the expression of the angiotensin-converting enzyme 2 (ACE2) surface receptor, the receptor for SARS-CoV-2 human cell binding, in several tissues such as muscle, cartilage, bone, and synovium (9). Rheumatic musculoskeletal manifestations of COVID-19 can be classified as rheumatic manifestations: (i) occurring during the infection; (ii) due to the medications used for the treatment of COVID-19; (iii) following vaccine administration; and (iv) caused by post/long COVID-19.

The aim of this Research Topic was to collect original research and review manuscripts dealing with musculoskeletal manifestations that can be observed in COVID-19. Six manuscripts were accepted: three original research articles, one mini-review, and two hypothesis and theory articles. These articles all cover the current relevant issues on musculoskeletal complaints of patients with COVID-19: the interrelations between COVID-19 and an underlying inflammatory rheumatic disease (IRD); the factors that may be associated with severe COVID-19 in patients with IRD; how to explain the muscle and joint manifestations during COVID-19; how to explain the sustained musculoskeletal pain during long COVID-19.

- Roseti and Grigolo discussed the relationships between COVID-19 and musculoskeletal diseases, with a focus on the similarities between COVID-19 and arthritis, especially the occurrence of autoantibodies and/or autoimmune diseases such as COVID-19-associated arthritis, which resembles reactive arthritis or clinical features of rheumatoid arthritis or systemic lupus erythematosus.
- One relevant question is the susceptibility of patients with IRD to COVID-19 and, in particular, the risk of developing a severe form of the disease, taking into account the immune deregulation of these conditions and the immunosuppressive medications that patients receive, especially biologic agents (1, 2). Previous European cohorts were reassuring on these concerns, and some specific treatments have been associated with a risk of severe disease, i.e., glucocorticoids, mycophenolate mofetil, and certain biologics such as rituximab (10). Cruz-Machada et al. reported the data from the Rheumatic Diseases Portuguese Registry and identified advanced age, comorbidities, and the use of rituximab as factors associated with an increased risk of infection and a worse prognosis, while Tumor Necrosis Factor (TNF) inhibitors and tocilizumab were associated with a reduced risk of infection. These data were in line with previous reports. In the same way, the CovAID study performed by Chevalier et al. aimed to identify the factors associated with severe COVID-19 in patients with autoimmune/inflammatory rheumatic diseases (AIRD). This was a large analysis performed on a French multicenter COVID-19 cohort using the database from the French national health insurance. Based on this analysis, treatment with rituximab or glucocorticoids and having vasculitis, autoinflammatory disease, or sarcoidosis were identified as factors associated with a higher risk of severe COVID-19. In general, patients with AIRD were more susceptible to severe COVID-19 as compared to the healthy population.
- Various bone manifestations may occur during COVID-19, such as osteonecrosis, heterotopic ossifications, and osteoporosis (OP) (11). Osteoporosis may be explained by glucocorticoid therapy administered to critically ill patients who required ventilation, experienced prolonged immobilization with sarcopenia, and suffered the effects of a cytokine storm on bone metabolism. The relationship between the development of OP and the severity of COVID-19 remains debated. In a Mendelian randomization study, Zhang et al. found no genetic causal link between COVID-19 severity and OP.
- Muscle is a leading target organ during SARS-CoV-2 infection, with various symptoms that include muscle weakness, myalgia, but also myositis, rhabdomyolysis, and sarcopenia (12). The mechanisms by which the skeletal muscle may be damaged by SARS-CoV-2 were analyzed by Veronesi et al., with a special focus on the interactions between the virus, the ACE2 receptor expressed on muscle cells, but also hypoxia and the cytokine storm.
- Finally, a large number of patients still presented with sustained symptoms after COVID-19. The WHO definition of post-COVID-19 is a condition that occurs in individuals with a history of probable or confirmed SARS-CoV-2 infection, usually 3 months after the onset of COVID-19, that persists for at least 2 months and cannot be explained by an alternative diagnosis (11). Long/post-COVID-19 symptoms include various symptoms such as fatigue, widespread pain, weakness, and muscle pain. This condition still remains unexplained, and it has been suggested that muscle denervation, deconditioning, sarcopenia, immune myopathy induced by the virus, and nutritional deficiency may explain in part these clinical features (9, 13). Central sensitization has also been proposed to explain the diffuse pain. Plaut examined this specific condition and reported that the so-called “fascial armoring” hypothesis that is proposed in fibromyalgia may be extended to long COVID-19, pointing to mechanical abnormalities in soft tissues, especially the fascia, with the active participation of myofibroblasts.

Overall, we believe that this Research Topic is a valuable contribution to the knowledge of the musculoskeletal involvement of COVID-19, and we trust that it will be useful for researchers and physicians caring for patients with COVID-19.

## Author contributions

ET: Writing—original draft. MM: Writing—review and editing. MF: Writing—review and editing. FS: Supervision, Writing—review and editing.
